# Sexual jokes at school and psychological complaints: Student- and class-level associations

**DOI:** 10.1177/1403494820974567

**Published:** 2020-12-07

**Authors:** Sara Brolin Låftman, Ylva Bjereld, Bitte Modin, Petra Löfstedt

**Affiliations:** 1 Department of Public Health Sciences, Centre for Health Equity Studies (CHESS), Stockholm University, Sweden; 2 Department of Behavioural Sciences and Learning (IBL), Linköping University, Sweden; 3 Department of Public Health and Community Medicine, University of Gothenburg, Sahlgrenska Academy, Sweden

**Keywords:** Sexual harassment, school, students, health complaints, contextual, multilevel

## Abstract

*Background:* Students who are subjected to sexual harassment at school report lower psychological well-being than those who are not exposed. Yet, it is possible that the occurrence of sexual harassment in the school class is also stressful for those who are not directly targeted, with potential negative effects on well-being for all students. *Aim:* The aim was to examine whether exposure to sexual jokes at the student level and at the class level was associated with students’ psychological complaints, and if these associations differed by gender. *Method:* Data from the Swedish Health Behaviour in School-aged Children (HBSC) of 2017/18 was used, with information from students aged 11, 13 and 15 years (*N*=3720 distributed across 209 classes). Psychological health complaints were constructed as an index based on four items. Exposure to sexual jokes at the student level was measured by one item, and at the class level as the class proportion of students exposed to sexual jokes, in per cent. Two-level linear regression analyses were performed.*Results:* Students who had been exposed to sexual jokes at school reported higher levels of psychological complaints, especially boys. Furthermore, the class proportion of students who had been exposed to sexual jokes was also associated with psychological complaints, even when adjusting for student-level exposure to sexual jokes, gender, grade and class size. ***Conclusions:* Sexual jokes seem to be harmful for those who are directly exposed, but may also affect indirectly exposed students negatively. Thus, a school climate free from sexual jokes may profit all students**.

## Introduction

Sexual harassment is a common problem in schools, and therefore often normalised by students [1]. According to the Swedish Discrimination Act [2], sexual harassment refers to actions of a sexual nature that violate someone’s dignity, and is classified as a form of discrimination. Sexual harassment may be expressed through various types of behaviour, including verbal (e.g. demeaning comments about gender and sexualised conversations), non-verbal (e.g. sexualised contact seeking and sexual looks) and sexual-assault behaviours (e.g. grabbing or pinching, or touching private body parts) [3,4]. Verbal sexual harassment is more prevalent than physical [5,6]. Sexual jokes are one type of verbal sexual harassment. Students who are exposed to sexual jokes often have a hard time defending themselves. Although being the object of sexual jokes can evoke feelings of anger, frustration and being hurt, a negative response to the joke can be further used as an accusation of lacking a sense of humour [7].

Already among elementary school students (grades 1–6), verbal, non-verbal and sexual assault behaviours are prevalent in the school setting [4]. In a Swedish study of grade 9 students, about 26% of girls and 14% of boys responded that they had received unwanted comments about their body at some point, and 34% of the girls and 5% of the boys reported that they had been touched or pawed against their will [8].

A number of studies have reported that students who are exposed to sexual harassment report poorer psychological health [8–15]. Yet, those who are not directly targeted may also perceive the presence of sexual harassment as a problem. It is more common for students to witness peers being sexually harassed than to be exposed themselves [6]. A study performed among girls in upper secondary school reported that sexual harassment was perceived as a (sometimes severe) problem both by students who were directly exposed and by those who were not [3]. Thus, it is possible to assume that witnessing or knowing about sexual harassment in the class is also perceived as stressful and as a threat among those who are not directly targeted, with potential implications for their psychological well-being. One study conducted among middle school students did not find any association between witnessing sexual harassment and psychological outcomes [6]. Nevertheless, studies of sexual harassment in the adult workplace have shown that the indirect exposure to sexual harassment, in terms of the general level of sexual harassment in a work group, is associated with poorer health outcomes among both women [16] and men [17]. There is, however, a scarcity of studies analysing contextual effects of sexual harassment among students.

Prior research has reported that girls are more exposed to sexual harassment than boys are [8,14,15], although some studies have found no difference between genders [12]. Furthermore, several studies have shown that the association between sexual harassment and poor psychological health is especially pronounced among girls [8,12], while others found that the strength of the association between sexual harassment and poor psychological health was similar across genders [14].

The aim of the current study was to examine whether exposure to sexual jokes at the student level and at the class level was associated with students’ psychological complaints, and if these associations differed by gender.

## Methods

Data from the Swedish Health Behaviour in School-aged Children (HBSC) of 2017/18 was used, with information collected among students in grades 5, 7 and 9, corresponding to the approximate ages 11, 13 and 15 years [18]. The HBSC study has been conducted regularly in Sweden since 1985/86 and is carried out in a large number of countries as part of a collaborative World Health Organization project. The purposes of the HBSC study are to increase knowledge about the social determinants of young people’s health and well-being, to analyse the development over time and to compare the situation of adolescents in different countries. The survey covers a broad range of indicators. The Swedish 2017/18 survey included a battery on bullying victimisation, of which one item addressed exposure to sexual jokes [18].

In Sweden, children enrol in compulsory school in a preschool class the year that they turn six years of age, and subsequently attend grades 1–9 at ages 7–16 years. Students normally belong to a specific school class unit, which consists of a group of students who spend most of the instruction time at school together, although they attend certain lessons in constellations with other students (e.g. modern languages).

The sampling and the data collection were performed by Statistics Sweden. For each grade, a two-step cluster sampling design was employed. In the first step, a random, nationally representative sample of schools was drawn. In the second step, one class was randomly drawn in each school that had agreed to participate. In total, 450 schools were included in the sampling frame, of which 213 (47%) agreed to participate (Public Health Agency of Sweden (2019), table 1.1) [18]. All students in that class were invited to participate. They were informed about the background and rationale of the study, that participation was voluntary, that they were also free to skip questions and that their responses were anonymous and could not be linked to any identified student or school. The participants completed the questionnaires with pencil and paper in the classroom, and returned the questionnaires to the teacher in sealed envelopes. The participation rate among students in the schools that agreed to participate was 89% (grade 5: 88%; grade 7: 90%; grade 9: 87%; Public Health Agency of Sweden (2019), Table 1.2) [18]. Combining the attrition at the school level (i.e. attrition of schools that were included in the sample but did not participate) and at the student level (i.e. attrition of students who attended schools that participated but who were absent during the day of the survey) rendered a total response rate of 42% [18]. In the 2017/18 survey, 4294 students participated. For the analyses of the present study, we omitted students in classes with fewer than eight students in order to create a reliable class-level measure of sexual jokes (*n*=25). Individuals with non-response on any of the included variables were also excluded (*n*=549). This resulted in a study sample of 3720 students distributed across 209 school class units. Because the students were anonymous, no formal approval from an ethical review board was required. More information about the data collection as well as the complete questionnaire (in Swedish) is provided by the Public Health Agency of Sweden [18].

### Measures

Psychological complaints were based on the question ‘How often during the past six months have you had the following complaints?’, and the response options ‘felt low’, ‘felt irritable or bad tempered’, ‘felt nervous’ and ‘had difficulties in getting to sleep’. The response categories were based on a five-point Likert scale, ranging from ‘practically every day’ to ‘seldom or never’, and were assigned values from 5 to 1. The four items had good internal consistency (Cronbach’s α=0.78). The items were summed to an index which had a range of 4–20, with higher values indicating more psychological complaints. Using data from the Canadian HBSC, Gariepy et al. [19] reported good internal and external construct validity of these four items, concluding that a scale based on these items constitutes a good measure of adolescents’ psychological health.

Exposure to sexual jokes at the student level was measured by one item concerning bullying at school: ‘Other students have exposed me to sexual jokes’. The response categories were: ‘I have never been bullied’, ‘I have been bullied once or twice’, ‘I am bullied two or three times a month’, ‘I am bullied about once a week’ and ‘I am bullied several times a week’. Students who marked that this happened at least two or three times a month were classified as exposed to sexual jokes at school.

Exposure to sexual jokes at the class level was defined as the class proportion of students exposed to sexual jokes, reported in per cent.

Gender had two categories: boy and girl.

Grade indicated whether the student attended grade 5, 7 or 9.

Class size indicated the number of responding students in each class.

### Statistical method

Since the students were nested in school classes and the aim was to investigate the associations between exposure to sexual jokes at the student class level and students’ psychological complaints, two-level random intercept linear regression analyses were performed using the ‘mixed’ command in Stata v15 (StataCorp, College Station, TX) [20]. Six separate models were performed. Model 1 included gender, grade and exposure to sexual jokes at the student level. Model 2 added the class proportion of students exposed to sexual jokes and class size. Model 3 included the interaction term for student-level exposure to sexual jokes and gender, whereas model 4 instead tested for the interaction between class-level exposure to sexual jokes and gender. Finally, we performed stratified analyses for boys and girls, respectively, including student- and class-level exposure to sexual jokes, grade and class size. For all models, the intra-class correlation (ICC) is reported. The ICC indicates the amount of variation in the dependent variable that can be attributed to the higher level (here, the class level), and is reported in per cent.

## Results

Descriptive statistics of the data are presented in Table I.

**Table I.** Descriptives (*N*=3720).

			Min	Max
*Student level*				
Psychological complaints	*M*=10.5	*SD*=4.0	4	20
Exposed to sexual jokes, *n* (%)				
No	3533	95.0		
Yes	187	5.0		
Gender, *n* (%)				
Boys	1814	48.8		
Girls	1906	51.2		
Grade, *n* (%)				
5	981	26.4		
7	1246	33.5		
9	1493	40.1		
*Class level*				
Proportion (%) of students in class who have been exposed to sexual jokes	*M*=5.3	*SD*=6.7	0	44.4
Class size	21.9	5.0	8	34

Proportions and numbers of students who reported having been exposed to sexual jokes, by gender and grade, are presented in Table II. Exposure to sexual jokes was reported by 3.9% of boys and 1.8% of girls in grade 5, by 5.2% of boys and girls in grade 7 and by 6.2% of boys and 6.4% of girls in grade 9. Chi-square tests show that there were no statistically significant gender differences.

**Table II.** Exposure to sexual jokes, by gender and grade.

	Boys (*N*=1814)		Girls (*N*=1906)		χ^2^	*p*
	*n*	%	*n*	%		
Grade						
5	19	3.9	9	1.8	3.61	0.057
7	32	5.2	33	5.2	0.00	0.985
9	44	6.2	50	6.4	0.02	0.881
All	95	5.2	92	4.8	0.33	0.567

Next, we performed a set of two-level regression analyses in order to assess the associations between the individual and school class measures of sexual jokes and psychological complaints, with results presented in Table III. Model 1 includes exposure to sexual jokes at the student level, as well as gender and grade. Exposure to sexual jokes was associated with a higher level of psychological complaints (*b*=2.89, *p*<0.001). Furthermore, girls reported higher levels of psychological complaints than boys did (*b*=2.04, *p*<0.001). Compared to students in grade 5, the level of psychological complaints was higher among students in grade 7 (*b*=0.62, *p*<0.01) and among those in grade 9 (*b*=1.53, *p*<0.001). In model 2, the proportion of students in the school class who had been exposed to sexual jokes was added, and was shown to be associated with higher levels of psychological complaints, even when adjusting for exposure to sexual jokes at the student level, as well as gender, grade and class size (*b*=0.03, *p*=0.015). Subsequently, the interaction between individual-level exposure to sexual jokes and gender was tested for (model 3). The interaction proved to be statistically significant, and indicated that the association between own exposure to sexual jokes and psychological complaints was stronger among boys than among girls. Next, in model 4, a cross-level interaction of the proportion of students in the school class who were exposed to sexual jokes and gender was included, but was not statistically significant. Finally, gender-separate analyses are presented for boys and girls. The results show that as indicated by the statistically significant interaction term reported in model 3, exposure to sexual jokes was more strongly associated with psychological complaints among boys (*b*=3.39, *p*<0.001) than among girls (*b*=2.01, *p*<0.001).

**Table III.** Psychological complaints by exposure to sexual jokes and proportion of students in class who have been exposed to sexual jokes.

	Model 1		Model 2		Model 3		Model 4		Boys (*N*=1814)		Girls (*N*=1906)	
	*b*	s.e.	*b*	s.e.	*b*	s.e.	*b*	s.e.	*b*	s.e.	*b*	s.e.
*Student level*												
Exposed to sexual jokes												
No (ref.)	0.00		0.00		0.00		0.00		0.00		0.00	
Yes	2.89***	0.28	2.74***	0.29	3.31***	0.40	2.74***	0.29	3.39***	0.38	2.01***	0.44
Gender												
Boy (ref.)	0.00		0.00		0.00		0.00					
Girl	2.04***	0.12	2.04***	0.12	2.10***	0.13	2.00***	0.16				
Grade												
5 (ref.)	0.00		0.00		0.00		0.00		0.00		0.00	
7	0.62**	0.21	0.52*	0.21	0.52*	0.21	0.52*	0.21	−0.04	0.26	1.16***	0.29
9	1.53***	0.20	1.38***	0.21	1.39***	0.21	1.38***	0.21	0.68**	0.25	2.14***	0.29
*Class level*												
% students in class exposed to sexual jokes			0.03*	0.01	0.03*	0.01	0.03	0.02	0.03*	0.02	0.03^†^	0.02
Class size			0.02	0.02	0.02	0.02	0.02	0.02	0.03	0.02	0.00	0.02
Exposed to sexual jokes*gender					−1.16*	0.56						
% students in class exposed to sexual jokes*gender							0.01	0.02				
ICC	4.0%		3.8%		3.7%		3.8%		4.3%		5.7%	

Unstandardised regression coefficients from two-level linear regressions. *N*=3720 students distributed across 209 classes.

*** *p*<0.001; **p<0.01; *p<0.05; ^†^*p*<0.10.

The class proportion of students who had been exposed to sexual jokes included a large number of zeros (i.e. students in school classes where no student reported having been exposed). Due to the skewed distribution of this class-level variable, we performed a sensitivity analysis using a categorical measure of the proportion of students who had been exposed to sexual jokes, with the results presented in Appendix Table A1. The reference category was classes with 0% students exposed to sexual jokes (40.7% of the sample; *n*=1515). The rest of the classes were split into two categories of about equal size, and captured classes with 1–7% students exposed to sexual jokes (30.0% of the sample; *n*=1114) and >7% students exposed to sexual jokes (29.3% of the sample; *n*=1091). The pattern shown in Table A1 is similar to that presented in Table III, but this sensitivity analysis also showed that the association between the class proportion of students who had been exposed to sexual jokes and psychological complaints was driven by classes where a relatively high proportion of students had been exposed.

## Discussion

This study demonstrated that exposure to sexual jokes at both the student and the class level was associated with higher levels of psychological complaints. While the association at the student level was strong and robust, the contextual level association was relatively weak.

The result that student-level exposure to sexual jokes was associated with higher levels of psychological complaints reflects and corroborates findings from previous studies showing that being subjected to sexual harassment was associated with poorer psychological health [8–15]. The association between student-level exposure to sexual jokes and psychological complaints was found to be stronger among boys than among girls. While previous studies have reported clearer associations between sexual harassment and psychological health among girls [8,12] or no gender differences in such associations [14], our finding may potentially be due to our focus on sexual jokes. The data contain no specification about the nature of these sexual jokes, but they could potentially include homophobic jokes and name calling. Homophobic name-calling victimisation and perpetration have been shown to be more prevalent among boys than among girls [14], and being exposed to such types of harassment may also be especially stigmatising for boys. Jokes about homosexuality have a powerful disciplinary mechanism for boys. To be called a ‘fag’ is to fail some of the masculine tasks of competence and heterosexual prowess, and instead reveal weakness [21]. In any case, it cannot be ruled out that the gender differences in the associations would likely have been different if we had also been able to capture other forms of verbal sexual harassment, as well as non-verbal acts of sexual harassment and sexual assault behaviour. Relatedly, it should be underlined that although verbal harassment is the most common type of sexual harassment [5,6], it is very likely that the prevalence rates and gender differences found in this study would have been different if other types of sexual harassment had been included. It should also be acknowledged that sexual jokes may differ in character. McMaster et al. [22] showed that same-gender and cross-gender sexual harassment perpetration constitute two types of aggressive behaviour, proposing that same-gender sexual harassment often involves homophobic insults and comments, whereas cross-gender sexual harassment is more likely to be related to (hetero-)sexual motives. They also found that the occurrence of same-gender perpetration did not vary by age, but that cross-gender perpetration was more common among students in grade 8 than in grade 6 [22]. Hence, with regards to the present study, it is possible that the character of the sexual jokes varied between the students in grades 5, 7 and 9, although the data did not allow us to investigate this empirically.

An interpretation of the finding that the prevalence of sexual jokes in the school class was positively associated with psychological complaints, even when adjusting for exposure to sexual jokes at the student level, is that the occurrence of sexual jokes in the school class is also a stressor for students who are not directly exposed. This result suggests that sexual jokes may be harmful not only to those who are directly targeted, but also to the student group as a whole. The result also reflects findings from studies of adult workplaces, which identified indirect exposure to sexual harassment as a predictor of psychological distress among both women [16] and men [17]. In addition, our findings regarding the class contextual associations of sexual jokes corroborate and extend the conclusions from prior research which has shown that the prevalence of bullying in a school class is associated with poorer psychological health among students, over and above the effects of exposure to bullying at the student level [23–27].

The main strength of the current study was that the hierarchical nature of the data enabled us to examine the associations between psychological complaints and sexual jokes at both the student and the class level. Furthermore, the fact that the data were based on a large, nationally representative sample of students in grades 5, 7 and 9 is a benefit, although the attrition, especially at the school level, implies that generalisations to these age groups in Sweden should be made with some caution. To be able to generalise the findings to a broader context, analyses of exposure to different types of sexual jokes at the student and the class level in relation to health outcomes should also be performed in other age groups and geographical settings.

There are, however, also limitations. As mentioned above, one limitation was that sexual harassment was measured by only one item capturing whether the students had been subjected to sexual jokes, implying that other forms of sexual harassment (i.e. other forms of verbal behaviour as well as non-verbal behaviour and sexual-assault behaviour) were not covered. Analysing both individual and contextual effects using more encompassing measures of sexual harassment is a promising task for future research. Furthermore, 11% of the students in the sampled classes did not participate in the study due to absence on the day of the survey or unwillingness to participate, and systematic bias among these non-responders cannot be ruled out. For instance, it is possible that students who are often exposed to sexual jokes and/or students with high levels of psychological complaints are more often absent from school and therefore less likely to have taken part in the study. However, we do not know whether any such bias exists and, if so, what that would imply for our findings. Another limitation concerns the cross-sectional nature of the data. Although it is likely that exposure to sexual jokes causes more psychological complaints, previous research on sexual harassment and psychological health outcomes based on longitudinal data has shown that the association exists in both directions, meaning that students with poor psychological well-being may be more vulnerable to sexual harassment [13]. Although there is less reason to question the directionality of the association between sexual jokes at the class level and students’ psychological complaints, future research should also assess this relationship by means of longitudinal data.

A final note concerns the fact that the current study could only differentiate between boys and girls, as there was no ‘neutral’ or ‘non-binary’ response category in the question on gender. One of the reasons why several students did not answer the question on gender (*n*=79) might have been because they did not identify as being either a boy or a girl. Since students with gender incongruence may be especially exposed to sexual harassment and are also more likely to report poorer psychological health than cisgender students [28], further research on individual and contextual effects of sexual harassment on health outcomes should also include this category of students.

## Conclusions

This study showed that students who were exposed to sexual jokes at school had higher levels of psychological complaints. Additionally, the proportion of students in the class who were exposed to sexual jokes was associated with higher levels of psychological complaints, over and above the association at the student level. These findings suggest that sexual jokes seem to be harmful for those who are directly exposed, but may also affect indirectly exposed students negatively. Thus, the study indicates that a school climate free from sexual jokes may benefit all students.

## Appendix

**Table A1.** Psychological complaints, by exposure to sexual jokes and proportion of students in class who have been exposed to sexual jokes (categorical measure).

	Model 1		Model 2		Model 3		Boys (*N*=1814)		Girls (*N*=1906)	
	*b*	s.e.	*b*	s.e.	*b*	s.e.	*b*	s.e.	*b*	s.e.
*Student level*										
Exposed to sexual jokes										
No (ref.)	0.00		0.00		0.00		0.00		0.00	
Yes	2.75***	0.29	3.30***	0.40	2.76***	0.29	3.41***	0.38	2.03***	0.43
Gender										
Boy (ref.)	0.00		0.00		0.00					
Girl	2.04***	0.12	2.10***	0.13	1.97***	0.19				
Grade										
5 (ref.)	0.00		0.00		0.00		0.00		0.00	
7	0.54**	0.21	0.55**	0.21	0.55**	0.21	-0.01	0.25	1.17***	0.29
9	1.33***	0.21	1.34***	0.21	1.34***	0.21	0.66*	0.25	2.09***	0.29
*Class level*										
% students in class exposed to sexual jokes										
0% (ref.)	0.00		0.00		0.00		0.00		0.00	
1–7%	−0.01	0.20	0.00	0.20	0.03	0.25	0.01	0.25	0.02	0.28
>7%	0.55**	0.20	0.55**	0.20	0.39	0.25	0.47^†^	0.25	0.63*	0.28
Class size	0.02	0.02	0.02	0.02	0.02	0.02	0.03	0.02	0.00	0.02
Exposed to sexual jokes*gender			−1.11*	0.56						
% students in class exposed to sexual jokes*gender										
1–7%*gender					−0.07	0.29				
>7%*gender					0.31	0.30				
ICC	3.6%		3.5%		3.6%		4.3%		5.3%	

Unstandardised regression coefficients from two-level linear regressions. *N*=3720 students distributed across 209 classes.

*** *p*<0.001; ***p*<0.01; **p*<0.05; ^†^*p*<0.10.

ICC: the intra-class correlation.
